# Effects of an Inhibitor of Monocyte Recruitment on Recovery from Traumatic Brain Injury in Mice Treated with Granulocyte Colony-Stimulating Factor

**DOI:** 10.3390/ijms18071418

**Published:** 2017-07-02

**Authors:** Shijie Song, Xiaoyuan Kong, Sandra Acosta, Vasyl Sava, Cesar V. Borlongan, Juan Sanchez-Ramos

**Affiliations:** 1 James A Haley VAH, Research Service, 13000 Bruce B. Downs Blvd, Tampa, FL 33612, USA; xkong@health.usf.edu (X.K.); vsava@health.usf.edu (V.S.); 2 Department of Neurology, University of South Florida, 13220 Laurel Drive, Tampa, FL 33612, USA; 3 Department of Neurosurgery and Brain Repair, University of South Florida, 12901 Bruce B, Downs Blvd, Tampa, FL 33612, USA; sacosta@health.usf.edu (S.A.); cborlong@health.usf.edu (C.V.B.)

**Keywords:** granulocyte-colony stimulating factor, monocyte chemotactic protein-1, radial arm water maze, bone marrow transplantation, hippocampal neurogenesis

## Abstract

Administration of the hematopoietic growth factor granulocyte-colony stimulating Factor (G-CSF) has been reported to enhance recovery from controlled cortical impact (CCI) in rodent models. G-CSF exerts actions in both the periphery (stimulation of hematopoiesis) and in the brain, where it serves as a neurotrophic factor, promoting neuronal survival and stimulating neural stem/progenitor cell proliferation in the hippocampus. In order to distinguish the direct CNS actions of G-CSF from its peripheral actions, experiments were designed to block the recruitment of peripheral monocytes to the site of the lesion produced by CCI. The selective C-C motif receptor 2 (CCR2) antagonist (RS504303) was co-administered with G-CSF for three days after CCI in a chimeric mouse previously transplanted with GFP-expressing (GFP+) blood stem-progenitor cells. Results: The drug significantly impaired infiltration of GFP+ bone marrow-derived cells to the frontal cortex and striatum without impeding recovery performance and hippocampal neurogenesis in the behavioral test, the Radial Arm Water Maze (RAWM). Administration of the CCR2 antagonist alone, without G-CSF, was effective in promoting recovery in RAWM. These results support the hypothesis that the direct action of G-CSF on neural cells, independent of its hematopoietic effects, is primarily responsible for enhanced recovery from CCI. In addition, this study confirms the importance of CCR2 and its ligand, monocyte chemotactic protein-1 (MCP-1), in mediating the inflammatory response following CCI.

## 1. Introduction

Granulocyte colony-stimulating factor (G-CSF) is one of several hematopoietic cytokines that regulates the production of circulating blood cells by the bone marrow. G-CSF is commonly used to treat leukopenia, but it has also been investigated in animal models of stroke. G-CSF administration was reported to reduce brain damage and improve functional outcomes [1,2,3,4]. G-CSF treatment has been shown to promote recovery from traumatic brain injury traumatic brain injury (TBI) in rodent models. Administration of intraperitoneal G-CSF (via osmotic minipump) in a rat model of TBI was shown to improve recovery of motor function compared to the control group [5]. In another study, intravenous (i.v.) administration of G-CSF (300 µg/kg) seven days after controlled cortical impact (CCI) to rats promoted transient motor benefits. In addition, G-CSF modestly increased hippocampal and subventricular zone (SVZ) neurogenesis and diminished microgliosis eight weeks after the TBI [6].

A recent study in mice examined the sub-acute response (up to two weeks after mild CCI) to a lower dose of G-CSF (100 µg/kg) given for three consecutive days after the injury [7]. That study was designed, in part, to determine the role and extent of infiltration into the brain of circulating monocytes, which serve to reinforce the microglial response to the injury. In order to track the transport and infiltration of monocytes, chimeric mice were generated by bone marrow transplantation of green fluorescent protein-expressing (GFP+) bone marrow stem cells into C57BL mice [7]. Following CCI to the right cerebral cortex, significant microgliosis and astrocytosis were observed in vehicle-treated mice, with the side of the trauma showing the greatest increase. G-CSF treatment increased astrocytosis on both sides of the brain, with the side of injury showing the greatest increase. G-CSF treatment also increased the extent of infiltration of GFP+ bone marrow-derived cells (BMDC). Approximately one third of the microglial signal (Iba1) overlapped with the GFP+ signal in the striatum on the side of the lesion by day 14 after CCI [7]. In addition, G-CSF treatment improved performance in a hippocampal-dependent learning task, the radial arm water maze (RAWM) on both days 7 and 14 after CCI. This behavioral improvement correlated with the enhanced expression of doublecortin (DCX), a surrogate index of hippocampal neurogenesis, in G-CSF-treated mice. The stimulation of neurogenesis is consistent with earlier reports that G-CSF enhanced neurogenesis in normal and AD mice [8,9] as well as in mice that had undergone CCI [6].

In addition to its impact on hematopoietic cells, G-CSF readily passes the blood–brain barrier [10] and interacts with G-CSF receptors, expressed by adult neural stem/progenitor cells, to promote neurogenesis [8,9,10,11]. G-CSF and its receptor are expressed by neurons in the CNS; their expression is induced by ischemia, implying an autocrine protective signaling mechanism [12]. G-CSF is now recognized to have multiple actions that contribute to long-term CNS plasticity. G-CSF exerts anti-apoptotic activity in mature neurons, triggers neuronal differentiation of adult neural stem cells in the brain, and promotes long-term recovery in more chronic stroke models [1,11,12].

To further understand the mechanisms for the beneficial effects of G-CSF in models of TBI, the present study was designed to determine the extent to which the recruitment of bone marrow-derived cells (BMDC), in particular monocytes, from the blood into the brain is responsible for enhanced recovery from TBI. The approach taken here utilized a selective chemokine receptor antagonist of monocyte chemoattractant protein-1 (MCP-1) to decrease the infiltration of monocytes into the CNS. If enhanced recovery from TBI occurs despite inhibition of monocyte recruitment to the site of injury, then the direct actions of G-CSF on neural cells can be considered a critical mechanism for enhanced functional recovery from TBI.

## 2. Results

**Figure 1:**
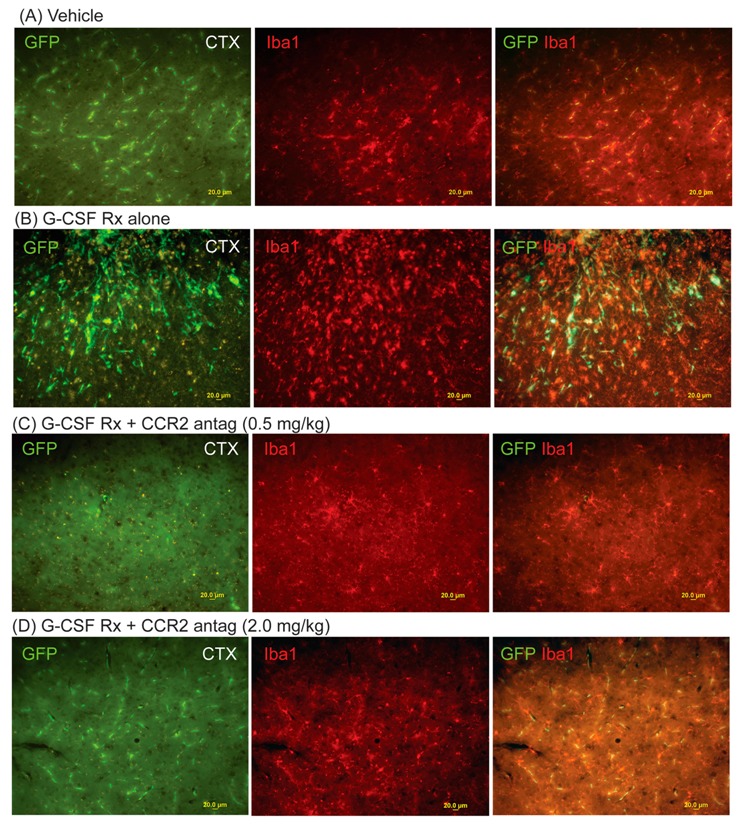
Effects of right-sided controlled cortical impact (CCI) on microgliosis (Iba1+ cells) and infiltration of green fluorescent protein-expressing (GFP+) cells into the right cerebral cortex at two weeks. The left-hand panels show GFP+ cells; middle panels show Iba1+ cells (microglia); right-hand panels show the composite image (GFP/Iba1). (**A**) Effects of vehicle treatment; (**B**) effects of granulocyte colony-stimulating factor (G-CSF) (100 µg/kg daily × 3 days) treatment after CCI. Note that many microglia co-express GFP+, indicating their origin from peripheral monocytes; (**C**) Effect of G-CSF and CCR2 antagonist treatment (0.5 mg/kg daily × 3 days); (**D**) effects of G-CSF and CCR2 antagonist treatment (2.0 mg/kg daily × 3 days) (scale bar = 20 µm).

**Figure 2:**
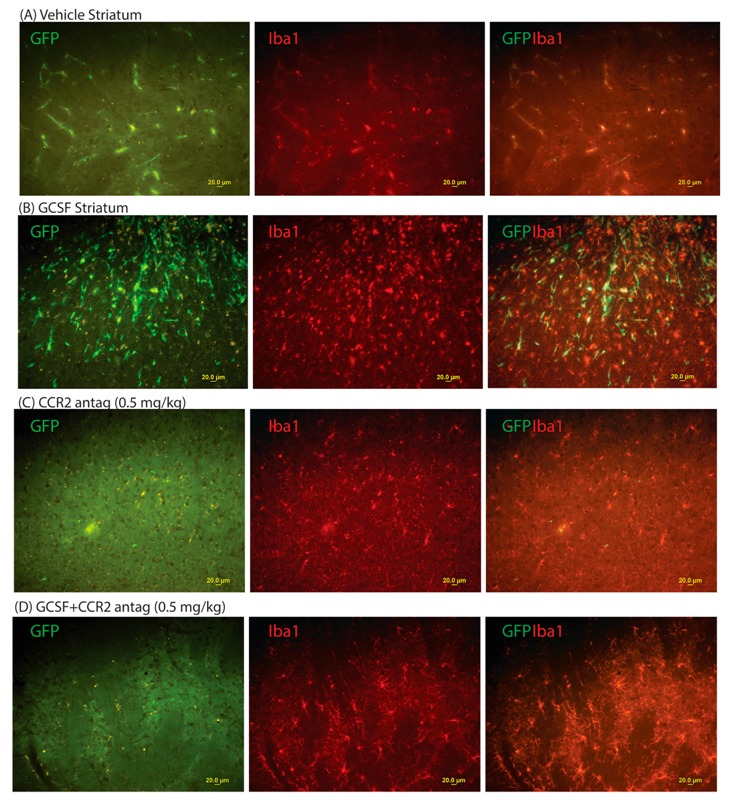
Effects of right-sided CCI on microgliosis (Iba1+ cells) and infiltration of GFP+ cells into the right corpus striatum at two weeks. Left-hand panels show GFP+ cells; middle panels show Iba1+ cells (microglia); right-hand panels show the composite image (GFP/Iba1). (**A**) Effects of vehicle treatment. Most of the GFP+ cells are within capillaries; (**B**) effects of G-CSF (100 µg/kg daily × 3 days) treatment after CCI. G-CSF increased the infiltration of GFP+ cells into the striatal parenchyma. Note that many microglia co-express GFP+, indicating their origin from peripheral monocytes; (**C**) effect of the CCR2 antagonist treatment alone (0.5 mg/kg daily × 3 days). Note that the CCR2 antagonist markedly diminishes the GFP+ signal, even in the capillaries of the striatum; (**D**) effects of G-CSF and CCR2 antagonist treatment (0.5 mg/kg daily × 3 days). Similar to the panels in (**C**), the CCR2 antagonist blocked the recruitment of GFP+ cells. The effects of the combination of G-CSF and 2.0 mg/kg CCR2 antagonist are not shown, but are similar to those produced by G-CSF and 0.5 mg/kg CCR2 antagonist as in Figure 2D (scale bar = 20 µm).

**Figure 3:**
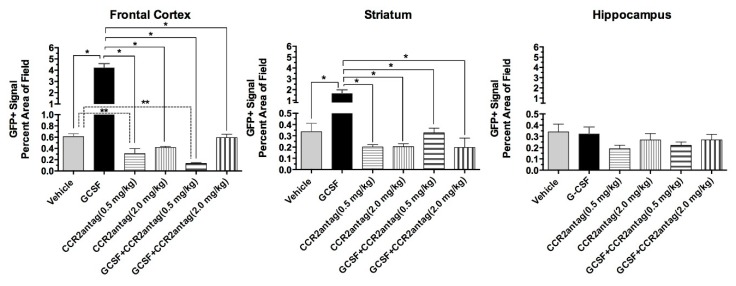
Summary data of GFP+ signal in the frontal cortex, striatum and hippocampus two weeks after CCI. The left panel shows an analysis of the GFP+ signal in the right frontal cortex. G-CSF-treated mice exhibited a significant increase in the GFP+ signal that was blocked by co-administration of the CCR2 receptor antagonist at both 0.5 and 2.0 mg/kg doses. One-way ANOVA (*p* = 0.0001) was followed by Sidak’s multiple comparison tests. * *p* < 0.05. It is noteworthy that the low dose CCR2 antagonist group alone significantly decreased the GFP+ signal compared to the vehicle-treated group. One-way ANOVA was run on all groups excluding the G-CSF group: multiple comparisons of the CCR2 antagonist (with and without G-CSF) compared to the vehicle-treated group revealed that the low dose CCR2 (0.5 mg/kg) groups exhibited a significant decrease in signal. ** *p* <0.05. The middle panel shows an analysis of the right striatum. G-CSF significantly increased the GFP+ signal compared to vehicle treatment. The GFP+ signal was blocked by co-administration of the CCR2 receptor antagonist at both doses. One-way ANOVA (*p* = 0.001) was followed by Sidak’s multiple comparison tests. * *p* < 0.05. The right panel shows an analysis of the right hippocampus. G-CSF did not increase the GFP+ signal. The administration of the CCR2 receptor antagonist alone tended to decrease the GFP+ signal compared to the vehicle-treated group, but this did not reach statistical significance.

Two weeks after CCI, the cohort of mice treated with G-CSF exhibited an increased infiltration of GFP+ (bone marrow-derived) cells into the right frontal cortex and striatum (Figure 1 and Figure 2). The GFP+ signal was reduced to a greater extent with the lower dose of CCR2 antagonist than the higher dose in the cortex (Figure 1C,D). In the striatum, the extent of the reduction of the GFP+ signal with the higher dose of CCR2 antagonist was the same as with the lower dose (0.5 mg/kg). See Figure 3.

Quantitative image analysis of the GFP+ signal revealed a six- and three-fold increase of the GFP+ signal in the right frontal cortex and the striatum, respectively. In addition, G-CSF treatment increased microglial activation in those regions (indicated by Iba1 immunolabeling) consistent with earlier reports [7,13]. Co-administration of both doses of CCR2 antagonist (0.5 and 2.0 mg/kg) blocked the infiltration of GFP+ to both the cortex and the striatum. One-way ANOVA followed by Sidak’s multiple comparison tests show that co-administration of the CCR2 antagonist with G-CSF resulted in significant decreases in the GFP+ signal compared to G-CSF treatment (*p* < 0.05) (Figure 3). When the effects of the CCR2 treatments (with and without G-CSF) were compared to vehicle treatment, only the low dose of the CCR2 antagonist was significantly different than the vehicle control (*p* < 0.05). The GFP+ signal in the hippocampus was not significantly increased two weeks following CCI compared to vehicle-treated controls (Figure 3).

**Figure 4:**
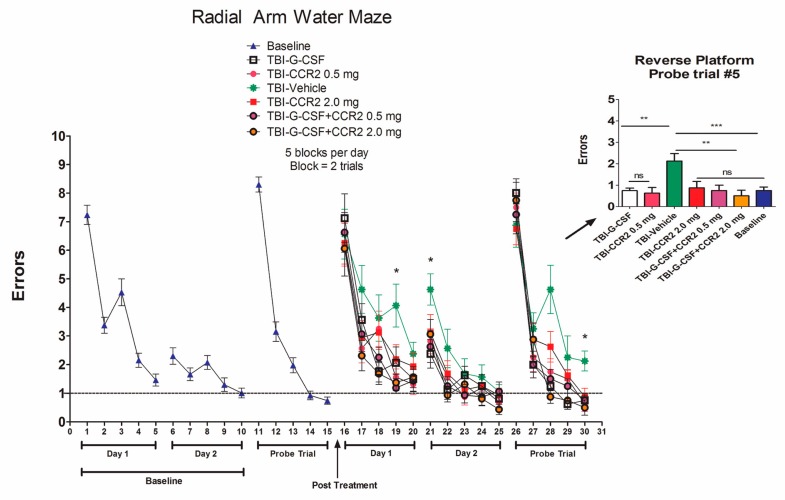
Effects of G-CSF and CCR2 antagonist treatment on performance in the Radial Arm Water Maze (RAWM). Summary data is plotted as the mean number of errors on the *y*-axis and trials on the *x*-axis. Baseline training on the RAWM was performed for three days before CCI. Drugs were then administered daily for three days after CCI. The RAWM was repeated on day 12 with reversal testing on day 14 post CCI (bar graphs). Asterisks indicate significant differences between treatments compared to each other and compared to baseline performance (* *p* <0.05), based on one-way ANOVA (*** *p* = 0.008) followed by Bonferroni multiple comparison tests (** *p* < 0.05).

G-CSF treatment improved performance in the RAWM compared to vehicle-treated controls (Figure 4). Interestingly, CCR2 antagonist co-administration with G-CSF did not prevent improved performance associated with G-CSF treatment. In fact, CCR2 antagonist treatment alone resulted in better performance in the RAWM compared to vehicle treatment.

**Figure 5:**
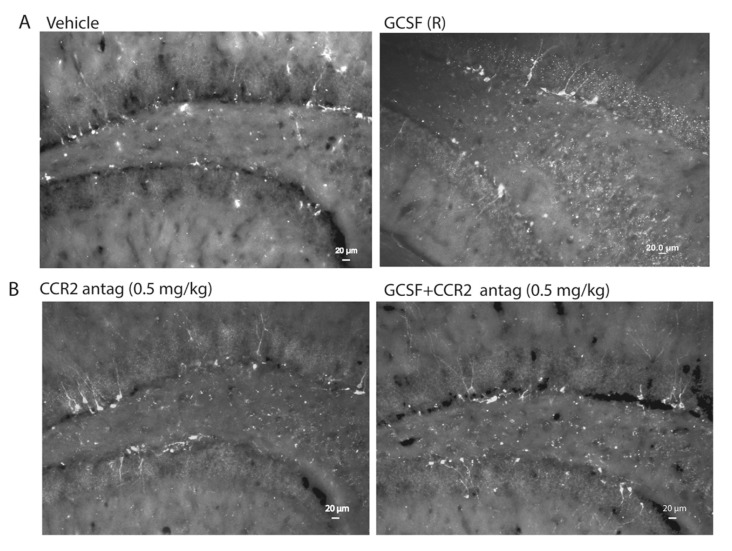

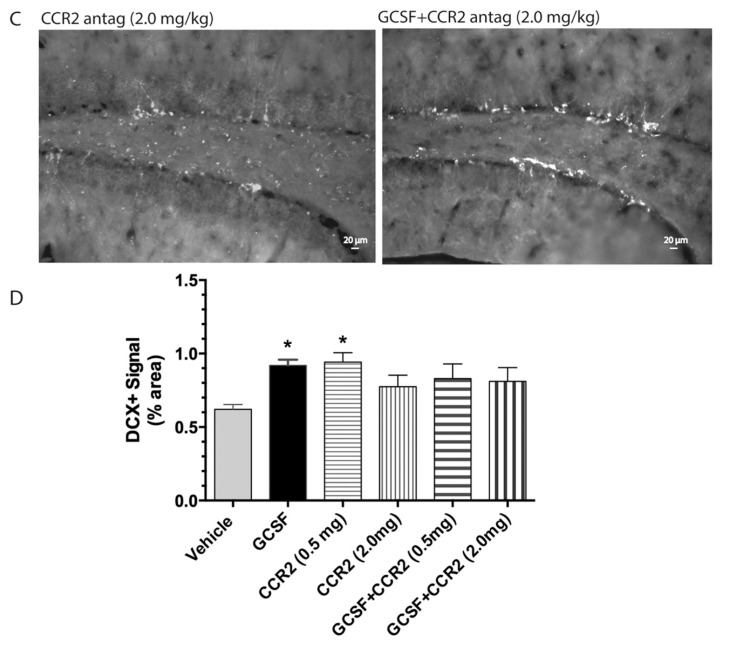
Effects of G-CSF and CCR2 receptor antagonist treatment on the expression of hippocampal doublecortin (DCX), a surrogate marker of neurogenesis. Arrows point to DCX+ cells in the subgranular zone. (**A**) Vehicle (**left panel**) compared to G-CSF treatment (**right panel**); (**B**) CCR2 receptor antagonist (0.5 mg/kg) alone (**left panel**) compared to G-CSF co-administered with the CCR2 receptor antagonist (0.5 mg/kg); (**C**) CCR2 receptor antagonist (2.0 mg/kg) alone (**left panel**) compared to G-CSF co-administered with the CCR2 receptor antagonist (2.0 mg/kg). Scale bar = 20 µm; (**D**) Summary of signal analysis. Mean ± SEM of the DCX+ signal (% of area) is plotted against specific treatment. Both G-CSF administered alone, and the CCR2 receptor antagonist administered alone increased the DCX signal in the subgranular zone of the hippocampus compared to vehicle treatment. One-way ANOVA was followed by Sidak’s correction for multiple comparisons (* *p* < 0.05).

G-CSF treatment, with or without CCR2 antagonist co-administration, triggered an increase in hippocampal DCX expression, a marker of immature neurons (Figure 5). In addition, the CCR2 antagonist administered alone at the low dose also significantly increased hippocampal neurogenesis (*p* < 0.05). DCX+ cell counts were not performed in this study because an earlier report from this laboratory showed a positive correlation between the DCX+ signal in the hippocampus and the DCX+ cell count in that structure [13].

## 3. Discussion

G-CSF administration (100 µg/kg daily × 3 days) promoted the recovery of performance in a hippocampal-dependent task assessed two weeks after CCI. The improved RAWM performance in the G-CSF treated cohort was associated with an increased recruitment of GFP+ bone marrow-derived cells to the site of the lesion and increased neurogenesis. This finding replicates earlier reports from this and other laboratories [5,6,7,13,14].

In addition to G-CSF actions in the periphery to increase circulating monocytes and increase their infiltration into CNS, G-CSF readily passes the blood–brain barrier [10] and interacts with G-CSF receptors, expressed by adult neural stem/progenitor cells, to promote neurogenesis [8,9,11]. G-CSF and its receptor are expressed by neurons in the CNS; their expression is induced by ischemia, implying an autocrine protective signaling mechanism [12]. G-CSF is now recognized to have multiple actions that contribute to long-term CNS plasticity. G-CSF exerts anti-apoptotic activity in mature neurons, triggers the neuronal differentiation of adult neural stem cells in the brain, and promotes long-term recovery in chronic models of stroke [1,11,12].

In order to distinguish the direct CNS actions of G-CSF from its peripheral actions, experiments were designed to block the recruitment of peripheral monocytes to the site of the lesion produced by CCI. The choice of the drug to block chemotaxis of monocytes was based on results from an in vitro study that showed an inhibitor of the chemokine receptor CCR2 (RS504303) was effective in blocking migration of monocytic cells across a fibronectin-coated micropore filter in vitro [15]. Hence, RS504393, the selective CCR2 antagonist was co-administered with G-CSF for three days after CCI. As reported here, the drug significantly impeded infiltration of GFP+ bone marrow-derived cells to the frontal cortex and striatum. In addition, both low and high doses of the CCR2 antagonist did not prevent recovery of performance from CCI. In fact, the performance in the RAWM following G-CSF was identical to the performance in the presence of the CCR2 antagonist. From these observations, it can be inferred that the direct actions of G-CSF in the CNS was sufficient to stimulate recovery from CCI and the infiltration of BMDC from the periphery was not necessary to improve performance in the RAWM after CCI.

Interestingly, cohorts of mice that were treated with the CCR2 antagonist alone performed as well as animals that received G-CSF alone. To understand this finding, it is important to clarify and discuss the chemokine system impacted by the drug. CCR2 (C-C motif receptor 2) is a G-protein-coupled receptor that binds to its natural ligand, the chemokine CCL2 (commonly known as monocyte chemoattractant protein-1 or MCP-1 [16]. MCP-1 mediates recruitment of inflammatory cells to sites of tissue injury [17,18]. MCP-1 has been reported to play an important role in the inflammatory response triggered by ischemia, traumatic brain injury, multiple sclerosis and excitotoxicity [19,20,21,22]. The receptor for MCP-1, CCR2, binds other chemokines, but MCP-1 is the major ligand and is considered to be the most potent in activating the signal transduction pathways that mediate monocyte recruitment [23]. The beneficial effects of CCR2 antagonism following TBI have previously been reported in a rat model [24]. Although the methodology and rodent species used by Liu et al was different, the findings reported here confirm the importance of the chemokine CCR-2/CCL-2 system in TBI. In the Liu et al study, local TBI in the adult rat cortex was induced by a weight-drop method. Expression of both MCP-1 and CCR2 in the tissue around the contusion site was markedly increased for at least 10 days after injury, peaking on day 3. The selective CCR2 antagonist, RS504393, decreased apoptosis and improved performance in the Morris water maze three days post-TBI, suggesting that CCL2–CCR2 signaling has deleterious effects on neuronal survival and learning [24]. Unlike the study by Liu et al.; the administration of the CCR2 antagonist enhanced hippocampal neurogenesis, an effect that correlates with improved performance in the RAWM, a hippocampal-dependent task. Mitigation of the inflammatory response with a low dose of MCP-1 following CCI produced effects very similar to the effect of G-CSF treatment alone.

## 4. Materials and Methods

This study was carried out in strict accordance with the recommendations from the Guide for the Care and Use of Laboratory Animals of the National Institutes of Health. The protocol was approved by the Institutional Animal Care and Use Committee at the University of South Florida. (IACUC # V4388, approved on 6 February 2015).

### 4.1. Animals

C57BL/6 mice, 8–10 weeks old, were purchased from Harlan Laboratories, and transgenic GFP mice (C57BL/6-*T*g [ACTB-EGFP] 1Osb/J, 003291) were obtained from Jackson Laboratory (Bar Harbor, ME, USA). All the experiments utilized chimeric mice, prepared from C57BL/6 mice transplanted with green fluorescent protein-expressing (GFP+) bone marrow. More specifically, the procedure for bone marrow harvesting from transgenic (*T*g) GFP+ mice has been previously published [7,9]. Briefly, bone marrow cells are collected from femurs and tibias of adult male GFP transgenic mice by flushing the bone shaft with PBS + 0.5% bovine serum albumin (BSA) + 2mM ethylenediaminetetraacetic acid (EDTA) (Sigma, St. Louis, MO, USA). To generate chimeric mice, C57BL/6J mice were lethally irradiated with 8 Gy total body irradiation (delivered in two fractions of 4 Gy, an interval of 4 h) at a dose rate of 1.03 Gy/min in a Gammacell 40 Exactor Following irradiation, the mice were given a bone marrow transplant (10 × 10^6^ mononuclear cells) from transgenic GFP mice infused via tail vein. Bone marrow-derived cells in the rescued mice were readily tracked by virtue of their green fluorescence. Examination of blood smears from tail clippings for the presence of green monocytes confirmed successful engraftment.

A total of 48 chimeric mice were prepared for this study. Cognitive performance in a hippocampal-dependent task (the radial arm water maze, or RAWM) was assessed at baseline, and 14 d after controlled cortical impact (CCI) in groups of eight mice. See Table 1 for distribution of the treatment groups.

### 4.2. Surgery and Controlled Cortical Impact

Animals underwent an experimental TBI using a controlled cortical impactor (Pittsburgh Precision Instruments Inc., Pittsburgh, PA, USA) as described previously [13,25]. Briefly, animals initially received Buprenorphine (0.05 mg/kg, s.c.) at the time of anesthesia induction (with 125 mg/kg Ketamine, 12.5 mg/kg Xylazine). After deep anesthesia was achieved (by checking for pain reflexes), individual animals were fixed in a stereotaxic frame (David Kopf Instruments, Tujunga, CA, USA). Craniectomy of exposed skull over the right frontoparietal cortex was performed (−0.5 mm anteroposterior and +0.5 mm mediolateral to bregma). This aperture accommodated the impactor tip of the pneumatically-operated TBI device (with a convex tip diameter = 2 mm), which impacts the brain at a velocity of 6.0 m/s reaching a depth of 0.5, 1.0 or 2.0 mm below the dura mater layer for mild, moderate and severe TBI, respectively, and remains in the brain for 150 ms [25]. For the purposes of the present study, a mild TBI was induced. The impactor rod was angled 15° to the vertical to maintain a perpendicular position in reference to the tangential plane of the brain curvature at the impact surface. A linear variable displacement transducer (Macrosensors, Pennsauken, NJ, USA), connected to the impactor, and measured velocity and duration to verify consistency. Bone wax was used to cover the craniectomized region and the skin incision sutured thereafter. A computer-operated thermal blanket pad and a rectal thermometer allowed maintenance of body temperature within normal limits. All animals were closely monitored until recovery from anesthesia and over the next three consecutive days.

### 4.3. Drugs

Human recombinant G-CSF (Neupogen^®^) was purchased from Amgen, Inc. (Thousand Oaks, CA, USA). The Neupogen was received in preservative-free vials containing 300 µg/mL. It was diluted to the appropriate concentrations in sterile 5% dextrose solution. The selective CCR-2 antagonist RS 504393 (Chemical Name: 6-Methyl-1′-[2-(5-methyl-2-phenyl-4-oxazolyl)ethyl]-spiro [4H-3,1-benzoxazine-4, 4′-piperidin]-2(1H)-one) was purchased from Tocris Biosci Inc. (Minneapolis, MN, USA). This chemokine receptor CCR-2 and its primary ligand, monocyte chemoattractant protein-1 (MCP-1), represent a critical signaling pathway for the recruitment of peripheral blood monocytes to sites of immune-mediated inflammation, where they become inflammatory macrophages.

### 4.4. Radial Arm Water Maze (RAWM)

The radial arm water maze (RAWM) task was employed to study the cognitive effects of G-CSF in mice that had undergone mild to moderate CCI. The RAWM is a hippocampal-dependent, spatial learning task that is not based on locomotor ability or swimming speed [26]. The RAWM was started on day 7 post-CCI. A six-arm radial arm maze was placed into a water tank with an approximately 100 cm diameter, and a 25 cm height, 5 cm diameter platform was used. The platform was submerged 0.5 cm below the water surface. The temperature of the water was kept at 26 °C. Mice were placed in the start arm at the beginning of every trial, and the platform was located in the goal arm. Every animal had an assigned platform/arm location throughout acquisition of learning, yet the starting zone was randomly changed per trial. A spatial-training protocol was followed. Mice were given two blocks of five trials, each block separated by a 30-minute rest period per day, for a total of 10 trials a day for two days of acquisition of learning for both baseline and post-TBI training. Trials were only 60 s long. Once animals found their goal arm/platform, they were allowed to remain on the platform for 30 s between trials. If mice were unable to find their goal arm/platform within 60 s, mice were guided to their goal arm and allowed to rest on the platform for 30 s. On day 3, a probe trial was given; this was reversal training in which the mice were placed 180 degrees from the goal arm. Mice were given five trials to train for the new position (reversal training). RAWM performance analysis was done by averaging the trials per block, using five trials per block, then a total of two blocks per day (errors are scored every time mice do not enter the goal arm).

### 4.5. Immunohistology

Mice were anesthetized with 150 mg/kg Ketamine, 15 mg/kg Xylazine and then transcardially perfused with 0.9% saline followed by 4% paraformaldehyde. Brains were stored in 4% paraformaldehyde, and then transferred to 25% sucrose solution in 4% paraformaldehyde, until the brains sank to the bottom. Then brains were slowly immersed into isopentane (cooled on dry-ice), left in isopentane for 20 s, removed, placed on a small piece of aluminum foil sitting on powdered dry-ice for 1–2 min (to let the isopentane evaporate) and finally wrapped in the foil and stored at −80 °C until sectioning. Brains slices were cut 30 μm thick, in a cryostat (Leica, Frankfurt, Germany) set to −25 °C.

Selective immunostaining of astrocytes and microglia was performed with antibodies to glial fibrillary acidic protein (GFAP) and ionized calcium-binding adapter molecule-1 (Iba-1), respectively. Iba-1 is protein that is specifically expressed in macrophages/microglia and is upregulated during the activation of these cells. Antibodies to doublecortin (DBX) were used to label immature neurons in the dentate gyrus of the hippocampus. Brain sections were preincubated in phosphate buffered saline (PBS) containing 10% normal serum (goat or donkey; Vector) and 0.3% Triton X-100 (Sigma) for 30 min. The sections were then transferred to a solution containing primary antibodies in 1% normal serum, 0.3% triton X-100/PBS and incubated overnight at 4 °C. The specific antibodies used in each experiment were: rabbit anti-DCX (Abcam Inc.; Cambridge, UK), 1:1000; 1:500; rabbit anti-Iba-1 (Wako Chemicals USA Inc., Richmond, VA, USA), 1: 500, rabbit anti-GFAP (BioGenex, Fremont, CA, USA), 1:50 in PBS containing 1:100 normal serum without Triton X-100. After incubation with a primary antibody, the sections were washed and incubated for one hour with Alexa Fluor 488 goat anti-mouse IgG diluted 1:400 in PBS (Invitrogen, Carlsbad, CA, USA) at room temperature. The sections were then rinsed in PBS three times and covered with a cover glass. Green fluorescence signals from the labeled cells were visualized with fluorescence microscopy using appropriate filters.

### 4.6. Quantitative Assessment of Bone Marrow-Derived Cells (GFP+ cells), Microglial Cells (Iba1+) and Hippocampal Neurogenesis (DCX+ Cells)

Quantitation of microgliosis (Iba1+ signal) and astrogliosis (GFAP+ signal) was made by computerized image analysis. An estimation of hippocampal neurogenesis (DCX+ signal) was also determined by image analysis. Previous reports from our laboratory documented a strong correlation between DCX+ signal analysis and stereologic counts of hippocampal DCX+ cells [13].

The method for quantitative image analyses has been previously described [9,27]. Eight mice per treatment group were analyzed at 14 days after CCI. Images were acquired at a magnification of 200X as digitized tagged-image format files (TIFF) to retain maximum resolution using an Olympus BX60 microscope with an attached digital camera system (DP-70, Olympus, Tokyo, Japan). Images of eight sections (each 30 microns thick and 180 microns apart) were captured from the serially sectioned striatum (approximately 1.2 mm in the AP dimension, starting at beginning of the lateral ventricles to the anterior commissure) on both the left and right side from each animal. Using ImageJ software (NIH, V1.48, Bethesda, MD, USA), the green channel was selected (to isolate the Alexa Fluor 488 signal) and converted into a monochrome signal. Then, a threshold optical density was obtained that discriminated staining from the background. Each anatomic region of interest was manually edited to eliminate artifacts. The thresholded signal was automatically generated by Image J and expressed as the total area of signal divided by the area of the 20X microscopic visual field (% visual field). Bias was eliminated by having the image analysis done by a blinded researcher.

### 4.7. Data Analysis and Statistics

Neurohistologic measures, as well as measures of neurotrophic factors were expressed as mean ± SEM and statistically evaluated using two-way ANOVA and multiple t-tests (comparing vehicle vs. G-CSF) with the Holm-Sidak correction for multiple comparisons (GraphPad version 6.01, LaJolla, CA, USA). Analysis of RAWM data utilized repeated measures analysis of variance (ANOVA) with one between-subjects factor (treatment) and one within-subjects factor (trials). One-way ANOVAs followed by Bonferroni’s post hoc test were also used to determine which trials were different between groups when a significant overall group difference or group × trial interaction was found. All comparisons were considered significant at *p* < 0.05.

## 5. Conclusions

In conclusion, the present study supports the hypothesis that the direct action of G-CSF on neural cells, independent of its hematopoietic effects, is primarily responsible for enhanced recovery from CCI. In addition, this study confirms the importance of CCR2 and its ligand MCP-1 in mediating the inflammatory response following TBI. More importantly, the administration of a drug that blocks MCP-1 actions (without the need for co-administration of G-CSF) appears to be sufficient to enhance recovery of cognitive performance in the RAWM. Going forward, it will be important to study the mechanism by which mitigation of the inflammatory response, by interfering with chemokine signaling, results in stimulation of the hippocampal neurogenesis.

## Figures and Tables

**Table 1:** Schedule of G-CSF and CCR-2 antagonist administration.

Group	Number of Mice	Treatments	Schedule
A	8	Saline + Saline	Daily × 3 days
B	8	Saline + G-CSF	Daily × 3 days
C	16	CCR-2 antag (0.5 or 2 mg/kg)+ Saline	Daily × 3 days
D	16	CCR-2 antag (0.5 or 2 mg/kg)+ G-CSF	Daily × 3 days

## Abbreviations

TBI	Traumatic brain injury
CCI	Controlled cortical impact
CCR2	C-C motif receptor 2
MCP-1	Monocyte chemoattractant protein-1
RAWM	Radial arm water maze
DCX	Doublecortin
CCI	Controlled cortical impact
GFP	Green fluorescent protein
